# People's knowledge, attitudes, practice, and healthcare education demand regarding OSA: a cross-sectional study among Chinese general populations

**DOI:** 10.3389/fpubh.2023.1128334

**Published:** 2023-07-13

**Authors:** Zhongjing Pan, Tianpei Ma, Qinghan Zeng, Ting Xu, Qiong Ran, Tianming Li, Dan Lu

**Affiliations:** ^1^Department of Otorhinolaryngology, Head, and Neck Surgery, West China Hospital, Sichuan University, Chengdu, China; ^2^Department of Epidemiology and Health Statistics, West China School of Public Health and West China Fourth Hospital, Sichuan University, Chengdu, China; ^3^Department of Outpatient Nursing, Dazhou Central Hospital, Dazhou, China; ^4^Department of Otorhinolaryngology, Head, and Neck Surgery, Santai People's Hospital, Mianyang, China

**Keywords:** OSA, general population, sleep health care education, KAP, public health

## Abstract

**Background:**

Population knowledge and attitudes toward obstructive sleep apnea (OSA) syndrome are critical to public health initiatives to overcome the disease. Healthcare education is an appropriate approach to expediting the process to build active medical practice models in the public.

**Objective:**

This study aimed to assess the level of knowledge, attitude, and practice (KAP) regarding OSA and healthcare education demand among the Chinese general population.

**Methods:**

A cross-sectional survey was performed online via Wenjuanxing in China between 8 February and 8 March 2022, using a 34-item questionnaire designed and reviewed by multidisciplinary experts.

**Results:**

This study enrolled 1507 respondents, aged 18 to 68, with a city-to-countryside ratio of approximately 2:1. Four-fifths of respondents reported that they had children (*n* = 1237), and mothers accounted for 57.7%. If they or their children had symptoms of OSA, nearly nine in 10 respondents would undertake positive medical practices, especially parents. A total of 89.4% of the respondents reported a desire to receive healthcare education through the new multimedia approach, and most were concerned about the etiology of OSA.

**Conclusion:**

The current study indicated that even the higher educated and urban populations in China had insufficient knowledge about positive attitudes toward and practices regarding OSA, indicating an urgent demand for healthcare education. A special emphasis should be placed on appropriating population demand for healthcare education and promoting the benefits of active medical practice models in sleep medicine.

## 1. Introduction

Obstructive sleep apnea (OSA) is a group of sleep breathing disorders involving both sexes and all ages. It is characterized by recurrent, intermittent upper airway obstruction, or reduced airflow during sleep, leading to repetitive episodes of desaturation, hypercapnia, and arousal (1). Adult patients with OSA typically complain of loud snoring, witnessed apnea, fragmented unrefreshing sleep, and daytime hypersomnolence. OSA is associated with a series of short-term and long-term negative consequences (2). If untreated, it not only can reduce patients' work efficiency and quality of life but also can relate to the occurrence and progression of chronic cardiovascular and metabolic diseases and neurocognitive problems and can increase the risk of all-cause mortality (3–5). The clinical presentation of children is different from that of adults, and pediatric patients with OSA often present with mouth breathing, nocturnal snoring and sweating, enuresis, and restless sleep (6, 7). Secondary to OSA, a myriad of negative consequences severely affect children's and teenagers' facial appearance and quality of life, such as adenoid face, poor school performance, possible depression, growth defects, and neurocognitive dysfunction (7–9).

The main risk factors for OSA are obesity and aging, which have increased in a striking proportion worldwide over the past four decades (10). Consequently, vulnerability to OSA has increased, affecting nearly one billion people or one in seven adults worldwide (11). Therein, the affected general population of China ranks at the top of the global list, with a prevalence of more than 23.6% among adults older than 30 years of age (11). However, as previous surveys have reported, the incidence of OSA has remained underestimated, and the undiagnosed proportion was 75 to 90% in the US (12, 13). In addition, the undiagnosed condition contributed to an enormous global healthcare burden, causing the social level costs to amount to more than $150 billion per year in the US (10, 14, 15), and medical costs up to €10.7 to €32.0 billion per year in Italy were due to OSA (16).

Considering the prevalence and negative effects of OSA on health, medical workers have paid more attention to OSA in various countries (17–19). Effective screening and early recognition performed by healthcare providers are needed to minimize the negative health impacts and maximize cost-effectiveness (11). The absence of awareness of the serious adverse effects of OSA among the general population results in people's unwillingness to be evaluated. However, previous studies have indicated that the current knowledge of and attitudes about OSA are insufficient among medical service providers (20–24). A survey performed in 2017 with a large sample of 1306 participants found that only 13.0% of participants could correctly define OSA (3). Xu et al. (25) conducted a descriptive study of childhood OSA in 2019 focused on parents in the Guangdong Province of China, suggesting that parents had limited awareness of its complications.

However, there is a paucity of data with large-scale samples focused on China regarding the knowledge, attitude, and practice (KAP) regarding OSA and healthcare demand among the general population. The purpose of this study was to evaluate the current KAP level and to investigate the education demand for OSA among the Chinese general population. In addition, we further explored the influencing factors for seeking medical help to determine whether there were correlations between the demographic data and medical practice patterns.

## 2. Methods

### 2.1. Participants

Before distributing the survey, the minimum sample size was calculated using G^*^Power (version 3.1; Heinrich Heine University) to achieve a power of 0.80. In the G^*^Power software, a logistic regression test was conducted for *a priori* power calculation with an odds ratio (OR) of 1.2 and a significance level of.05. The minimum sample needed to achieve a power of 0.80 was 1484 for our study. Considering the missing and non-responsive cases, we expanded it by 10%, yielding a predicted sample size of 1632. In the present study, the population focused on general adult residents who were from China, were at least 18 years old, and were competent to answer the questions online. Those who were illiterate or unwilling to participate were not invited. In the process of data screening, the inclusion criteria were Chinese residents ≥ 18 years old who volunteered to participate and were competent to comprehend the content of the questionnaire. Those who answered contradictory or factual content or had a response time of ≤ 180 s were excluded.

### 2.2. Ethics approval

Ethical approval regarding human subject research was obtained from the Ethics Committee on Biomedical Research, West China Hospital of Sichuan University (approval number: 2022.416). Informed consent was obtained from each participant online by placing a question about their agreement to participate in the study at the beginning of the survey. Participants were assured of the confidentiality and anonymity of this study and their rights to exit at any time. We declare that the data were collected for academic use only.

### 2.3. Instrument

The primary version of the questionnaire was developed in Chinese by an investigation team based on a deep literature review of comparable studies and international guidelines (3, 25–28), including otolaryngologists, medical sociologists, and a statistician. Researchers randomly invited 20 participants face-to-face from the general population to answer the questionnaire online for pretext and collected their feedback about the comprehensibility of questions and options. Four experts in the field of sleep medicine and survey studies reviewed these responses and each item of the survey and confirmed the final version of a 34-item questionnaire (see Supplementary material). The face validity and content validity of the survey were established. It comprised basic demographic data and four sections about knowledge, attitudes, practices, and demand regarding popular healthcare toward OSA. A series of options were listed with points for each question and zero for ignorance or mistakes. The total scores for symptoms, examinations, complications, and treatments in OSA were 13, 5, 12, and 9, respectively. Cronbach's α was >0.7 for each scale (0.885 for symptoms, 0.738 for examinations, 0.863 for complications, and 0.835 for treatments for OSA).

### 2.4. Procedures

**Figure 1:**
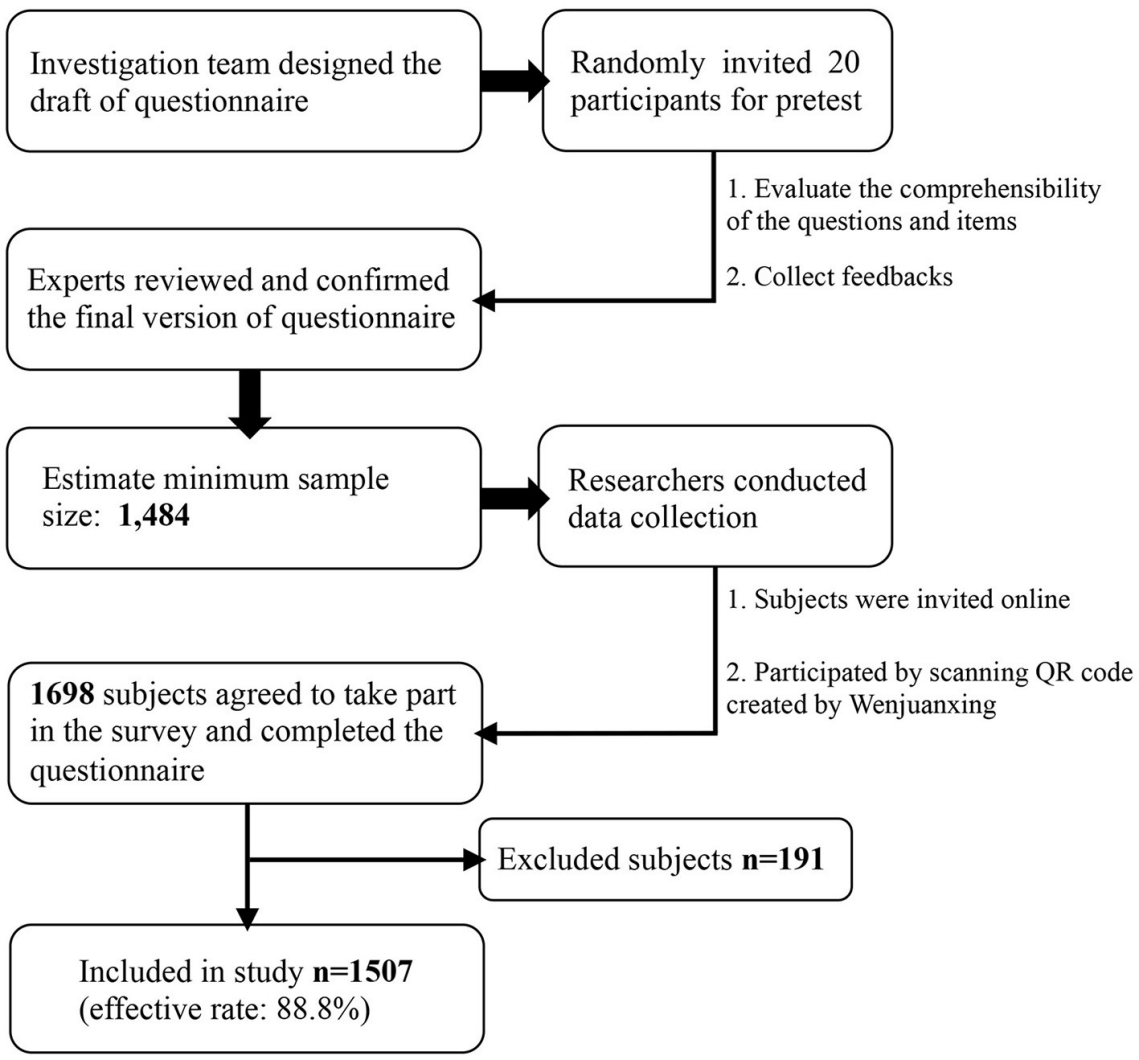
Basic flow chart of the study.

The cross-sectional survey was conducted in China between 8 February and 8 March 2022, using a 34-item questionnaire designed and reviewed by multidisciplinary experts (Figure 1). The general Chinese adult population was randomly invited online and offline. Participants were informed that the survey was based on voluntary principles and that their data would be anonymous and confidential. First, our investigation team created a questionnaire QR code (quick response code or dimensional barcode) by Wenjuanxing (https://www.wjx.cn), which is an online questionnaire platform widely used in academic studies in China. Then, researchers distributed the QR code using Chinese popular social media to get access to the general populations as many as possible, including WeChat (https://web.wechat.com) and QQ (https://www.qq.com). In addition, five researchers performed face-to-face invitations to scan the QR code in possible surveyed populations in different public scenarios, such as hospitals, universities, and commercial venues. Participants' IP addresses were restricted to ensure only one submission. The chief researcher was responsible for checking the collected data from Wenjuanxing, and up to 11.2% of submitted questionnaires were excluded for invalid response times and logistic errors.

### 2.5. Statistical analysis

The data are summarized as the means and standard deviations for continuous variables and percentages for categorical variables. Comparisons between groups were performed with the *t*-test and chi-square test. Univariate (unadjusted) and multivariate logistic regression models (adjusted for confounders) were applied to assess the associations between various demographic factors and the practice of seeking medical help for OSA. All statistical analyses were performed using IBM SPSS statistics software (version 26.0) and two-sided tests at a 5% level of significance (*p* < 0.05).

## 3. Results

### 3.1. Baseline characteristics of respondents

This study enrolled 1507 respondents (560 men and 947 women), their ages ranged from 18 to 68 years, and they came from 28 provinces, autonomous regions, and municipalities (including Sichuan, Zhejiang, Jiangsu, Liaoning, Fujian, Shandong, and Anhui provinces). The respondents' characteristics are shown in Table 1. Among employed respondents (*n* = 1,347), majors in medicine numbered 40.9%. Four-fifths of respondents reported that they had children (*n* = 1,237), mothers accounted for 57.7%, and fathers ranked second (36.5%).

**Table 1:** Participants' primary sociodemographic information.

**Sociodemographic**	**Data, *n* (%)**
Population	1,507
Age (mean ± SD)	36.2 ± 9.25
**Sex**	
Male	560 (37.2)
Female	947 (62.8)
**Region**	
Sichuan Province	878 (58.3)
Zhejiang Province	188 (12.5)
Jiangsu Province	105 (7.0)
Others	336 (22.2)
**Place of residence**	
Countryside	1,044 (69.3)
City	463 (30.7)
**Educational background**	
College degree and less	523 (34.7)
Bachelor's degree	810 (53.7)
Master's or doctoral degree	174 (11.5)
**Work status**	
Employed	1,347 (89.4)
Unemployed	160 (10.6)
**Monthly household income**	
< 10,000	947 (64.7)
10,000–30,000	338 (22.4)
≥ 30,000	195 (12.9)
**Whether they had children**	
Yes	1,237 (82.1)
No	270 (17.9)

### 3.2. Knowledge of OSA

When asked whether they had heard of OSA, 62.8% of respondents said they had only heard of it or knew little, and 292 respondents reported that they had never heard of it. Nevertheless, the majority regarded OSA as a severe disease (76.0%). In the description of OSA, only 37.2% of respondents answered all four questions correctly (Table 2). In particular, the description of “Untreated OSA would increase the morbidity of traffic accidents” at 68.5% correct was the lowest.

**Table 2:** Correct rate in four common descriptions of OSA.

**Description**	**Correct rate**
Obstructive sleep apnea syndrome increases the incidence of traffic accidents.	68.5%
The most common cause of obstructive sleep apnea in children is hypertrophy of the tonsils and adenoids.	72.5%
Children's hearing loss could be related to adenoid hypertrophy.	71.7%
Which picture shows an “adenoid face”?	71.9%

**Figure 2:**
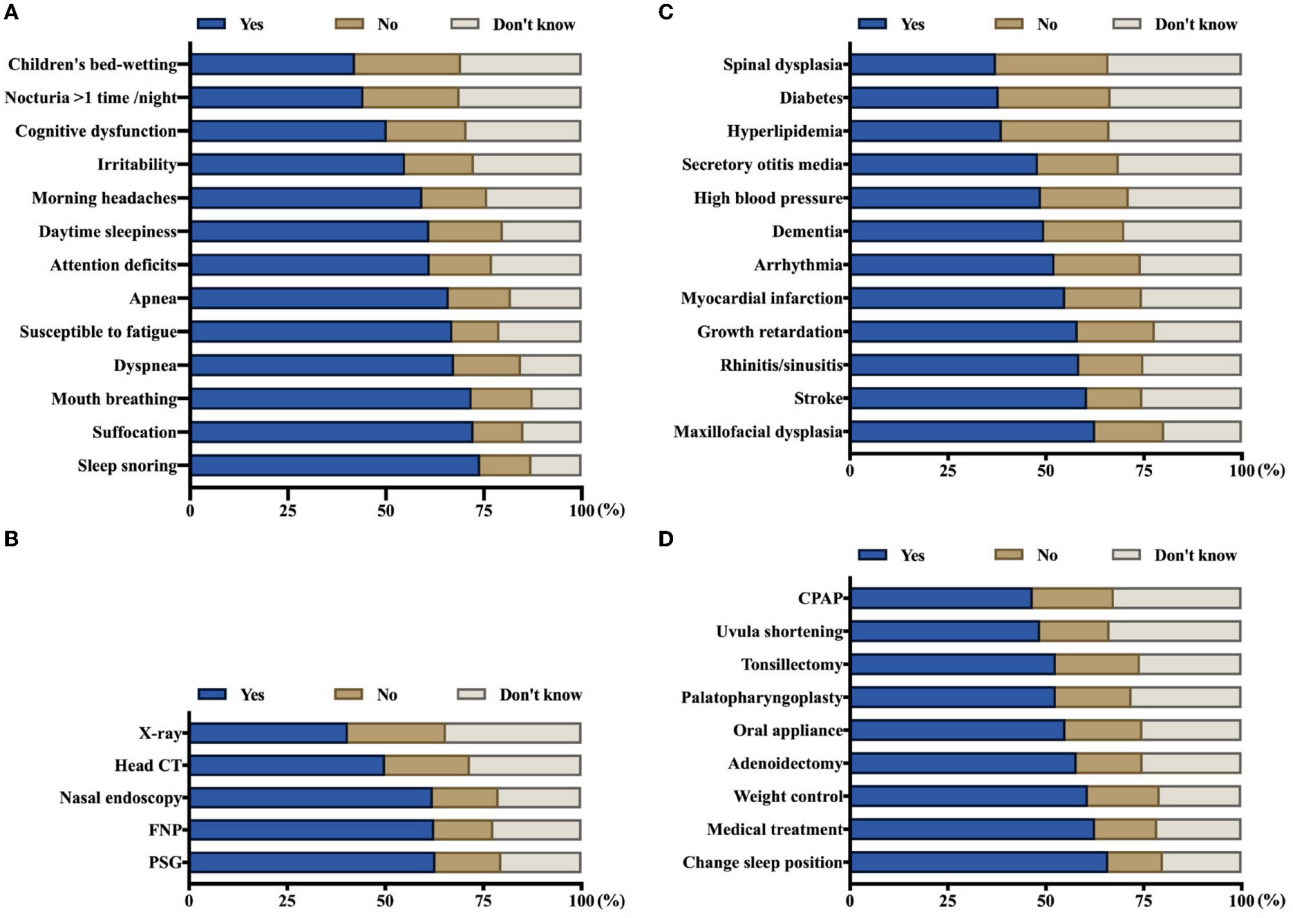
Awareness rate of symptoms, examinations, complications, and treatments among the general population, corresponding to parts **(A–D)** respectively (*N* = 1507). FNP, flexible nasopharyngoscopy; PSG, polysomnography; CPAP, continuous positive airway pressure.

Regarding diagnostic methods in OSA, the respondents were asked to identify the symptoms, examinations, and complications that those suffering from OSA might have, as shown in Figure 2. The average awareness rate of symptoms of OSA was the highest (60.9%), followed by the examination method (55.6%), and the complication rate was only 50.6%. In particular, nocturia more than once per night (44.3%) and bed-wetting in children (44.1%) were the most neglected symptoms of OSA. Approximately 40.5% of respondents could determine that X-rays are commonly used to diagnose OSA. Among its complications, <40% of respondents associated spinal dysplasia, diabetes, and hyperlipidemia with OSA. For the treatment of OSA, the average awareness rate was 55.9%, and continuous positive airway pressure was the most underrated treatment in the general public (46.7%) (Figure 2).

### 3.3. Attitudes and practices in OSA

When asked if they had symptoms of OSA and whether they sought medical help, most of the participants answered positively (87.6%), and they were most likely to visit the otolaryngology and respiratory medicine clinics. For parents, 95.6% of them indicated that they would take their children to see doctors if their child or children had related presentations (*n* = 1,237). A total of 73.3% of parents also chose to see otolaryngologists, only 39.5% of them chose pediatricians, and several participants referred to sleep medicine, departments of neurology, and traditional Chinese medicine. Regarding the reasons why they did not seek medical advice, more than half of them regarded OSA as a non-fatal disease, as shown in Table 3.

**Table 3:** Percentage of various reasons why they did not seek medical help when they had symptoms of OSA.

**Reasons for not seek medical help (*n =* 524)**	**Percentage (%)**
Obstructive sleep apnea is a non-fatal disease	54.4
Lack confidence for existing treatment options	40.2
Worry about surgery risks	38.3
During the epidemic of COVID-19, worry about the infection	33.2
Worry about medical cost	22.5
Have no time to see doctors due to the busy work	17.4
Worry about the time costs affecting children's learning	14.5
It is inconvenient to go to hospital	10.5
Other: ^a^	4.4

a Being unaware of the abnormal presentations or the disease had no influence on quality of life.

A total of 24.1% of respondents indicated they had a previous diagnosis of OSA, and 42.0% of parents reported that their children had been diagnosed with OSA. Among these children with OSA (*n* = 519), 42.2% had received conservative therapy, 28.1% had undergone surgical treatment, and 29.5% never had any intervention. Tonsillectomy and adenoidectomy were the most common surgeries they reported (65.8%, *n* = 146).

### 3.4. Healthcare education demand of OSA

Nine in 10 (89.4%) of the respondents were willing to receive popular healthcare education about OSA, and the most common referred content that they were eager to receive consisted of the causes, symptoms, complications, and treatment of OSA (74.9%). Some child caregivers reported that they wanted to differentiate OSA from benign snoring. Another wondered whether there were any effective methods to prevent this disease. In terms of the channels to access this healthcare information, the internet/new media and the healthcare delivery produced by medical professionals were the preferred outlets (68.3 and 67.0%, respectively).

### 3.5. Analysis of the factors influencing the KAP of OSA

**Figure 3:**
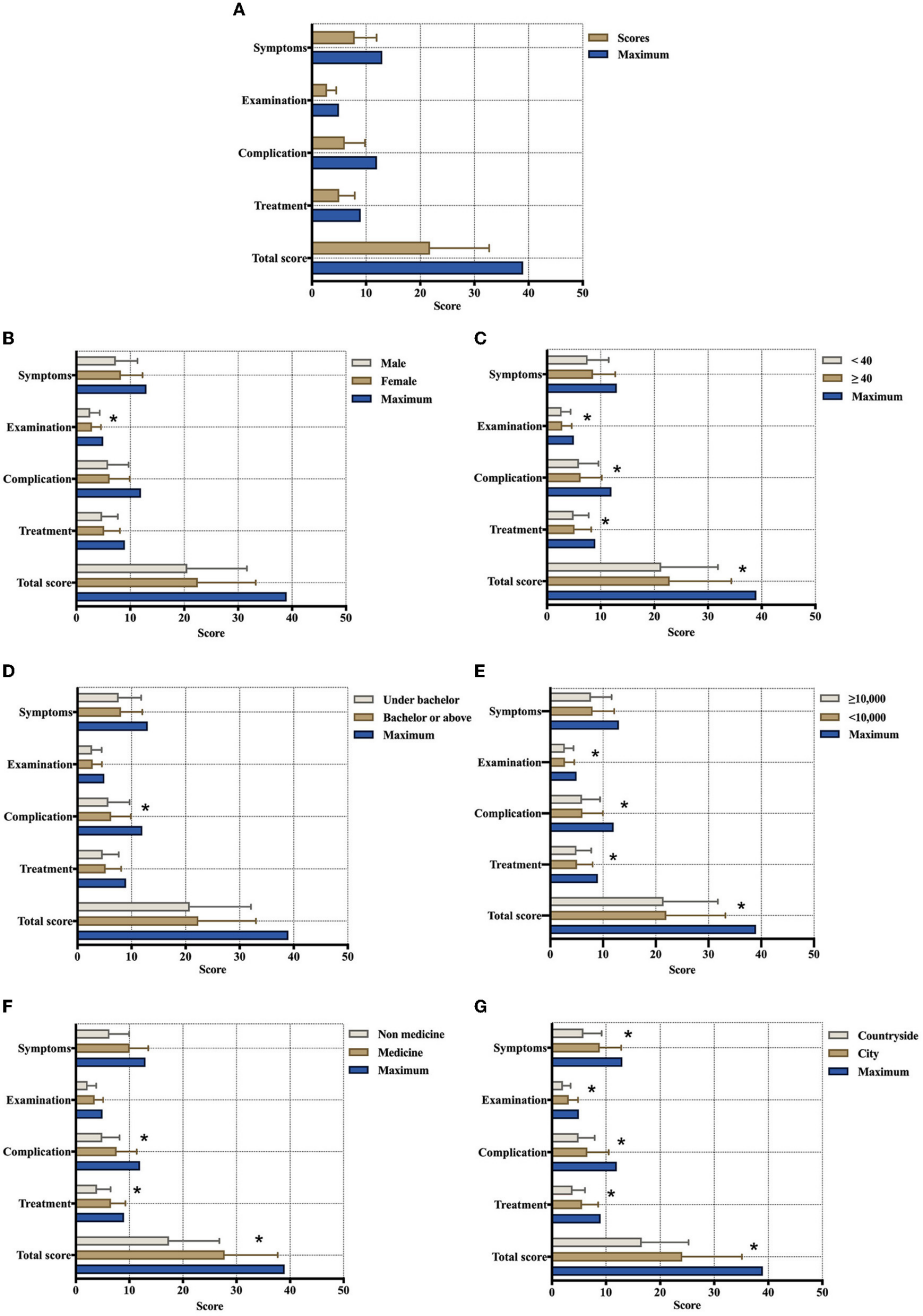
Various subgroups of scores in the symptoms, examinations, complications, and treatments of OSA among the general population. **(A)** Baseline full scores; **(B)** sex groups; **(C)** age groups; **(D)** educational groups; **(E)** monthly household income groups; **(F)** occupational groups (*n* = 1347); and **(G)** residential place groups (*N* = 1,507; *Indicates a significant difference).

In this study, the items of symptoms, examinations, complications, and treatments in OSA were accumulated scores as the baseline data. The comparison between these scores that showed significant differences in sex, age, educational background, household income, occupational background, and residence is shown in Figure 3. In the present study, respondents who were women, ≥ 40 years of age, had a bachelor's degree or more, had <10,000 yuan monthly household incomes, had a related medical occupation, and lived in a city with higher scores in OSA knowledge. The total scores of the four sections showed no significant differences in whether seeking medical advice when one's children had presentations of OSA (*p* = 0.085). To examine the influencing factors associated with seeking medical advice, the variables of interest were tentatively incorporated into a regression model (Table 4). In the multivariate regression, many demographic and individual factors independently influenced the practices regarding OSA among the general population. The results showed that the respondents with > 30,000 yuan monthly household incomes had a higher odds ratio for active practice regarding OSA (adjusted OR = 4.621, *p* = 0.001) compared to those in lower income groups (< 10,000 yuan). Additionally, the people who lived in the city had a higher odds ratio for seeking medical advice for OSA than those who lived in the countryside (adjusted OR = 2.789, *p* < 0.001). Respondents who had a prior diagnosis of OSA and had a positive attitude toward healthcare education were significantly more likely to seek medical advice on their own initiative (*p* < 0.001). In contrast, sex, age, educational and occupational background, and scores in the four sections of knowledge about OSA did not significantly affect their medical practice (*p* > 0.05).

**Table 4:** Factors associated with seeking medical advice in the general population when people had symptom**s** (logistic regression *N* = 1507).

**Covariable**	**UOR^a^ (95% CI^c^)**	***p*-value**	**ATOR^b^ (95% CI^c^)**	***p*-value**
**Gender**				
Male	1		1	
Female	1.040 (0.759–1.426)	0.807	1.189 (0.837–1.690)	0.334
**Age**	0.981 (0.966–0.997)	0.021	0.995 (0.978–1.011)	0.534
**Educational background**				
College degree and less	1	-	1	
Bachelor's degree	1.770 (1.272–2.465)	0.001	1.394 (0.969–2.006)	0.074
Master's or doctoral degree	1.074 (0.666–1.732)	0.771	0.985 (0.567–1.711)	0.956
**Occupational background**				
Non-medicine-related job	1		1	
Medicine-related job	1.133 (0.798–1.607)	0.485	1.498 (0.992–2.262)	0.055
**Monthly household income**				
< 10,000	1		1	
10,000-30,000	1.319 (0.904–1.925)	0.151	1.306 (0.868–1.966)	0.200
≥ 30,000	6.539 (2.644–16.173)	< 0.001	4.621 (1.825–11.701)	0.001
**Place of residence**				
Countryside	1	-	1	
City	2.555 (1.700–3.838)	< 0.001	2.789 (1.788–4.351)	< 0.001
Scores in symptoms of OSA	1.004 (0.967–1.043)	0.831	0.995 (0.934–1.059)	0.866
Scores in examination of OSA	1.044 (0.955–1.142)	0.341	1.047 (0.913–1.201)	0.512
Scores in complications of OSA	1.062 (1.018–1.080)	0.006	1.042 (0.974–1.118)	0.229
Scores in treatment of OSA	1.057 (1.002–1.115)	0.043	1.016 (0.929–1.112)	0.725
**Previous diagnosis of OSA**				
No	1		1	
Yes	6.461 (3.378–12.360)	< 0.001	4.872 (2.490–9.534)	< 0.001
**Demand for education in OSA**				
No	1		1	
Yes	3.321 (2.257–4.885)	< 0.001	2.301 (1.532–3.477)	< 0.001

a UOR is the abbreviation of unadjusted odds ratio.

b ATOR is the abbreviation of adjusted odds ratio.

c CI is the abbreviation of confidence interval (Adjusted OR, CI, and *p*-value per multiple logistic regression model with medical practice as the outcomes).

The subsequent analysis focused on the child caregivers group to further determine how demographic, knowledge, and attitude factors affected the practice of taking children to the clinic for OSA. In comparison to unemployed people, respondents who had stable work status (employed) were significantly more likely to undertake an active practice for OSA children (adjusted OR = 2.663, *p* = 0.03). Among the relationships with children, mothers had a higher odds ratio for taking children to seek medical advice for OSA than fathers (adjusted OR = 2.115, *p* = 0.017). Parents whose children had ever been diagnosed with OSA were significantly more likely to engage in positive practices for their children's symptoms (adjusted OR = 49.507, *p* < 0.001). Conversely, age, monthly household income, place of residence, the number of children < 14 years old, and scores in four sections of knowledge about OSA did not significantly affect the practice of treating OSA among child caregivers (*p* > 0.05) (Table 5).

**Table 5:** Factors associated with seeking medical advice in general populations when people's children had symptoms (logistic regression *n* = 1,237).

**Covariable**	**UOR^a^**	***p*-value**	**ATOR^b^**	***p*-value**
	**(95% CI^c^)**		**(95% CI^c^)**	
Age	0.940 (0.913–0.967)	< 0.001	0.989 (0.958–1.021)	0.5
**Work status**				
Unemployed	1		1	
Employed	4.962 (2.432–10.122)	< 0.001	2.663 (1.101–6.443)	0
**Monthly household income**				
< 10,000	1		1	
10,000–30,000	2.102 (0.976–4.527)	0.1	1.943 (0.867–4.358)	0.1
≥30,000	3.578 (1.098–11.666)	0	1.279 (0.362–4.521)	0.7
**Place of residence**				
Countryside	1	-	1	
City	0.512 (0.261–1.004)	0.1	1.331 (0.607–2.918)	0.5
**Relationship with children**				
Father	1		1	
Mother	1.490 (0.853–2.601)	0.2	2.115 (1.145–3.907)	0
Other	2.020 (0.468–8.720)	0.3	2.209 (0.391–12.463)	0.4
Have how many children ≤ 14 y	0.919 (0.836–1.011)	0.1	0.921 (0.841–1.009)	0.1
Scores in symptoms of OSA	1.029 (0.961–1.101)	0.4	0.994 (0.900–1.098)	0.9
Scores in examination of OSA	1.268 (1.069–1.503)	0	1.207 (0.953–1.527)	0.1
Scores in complications of OSA	1.146 (1.051–1.250)	0	1.041 (0.914–1.186)	0.5
Scores in treatment of OSA	1.200 (1.076–1.338)	0	1.105 (0.940–1.299)	0.2
**Children with a prior diagnosis as OSA**				
No	1		1	
Yes	41.284 (5.69–299.519)	< 0.001	49.507 (6.474–378.611)	< 0.001

a UOR is the abbreviation of unadjusted odds ratio.

b ATOR is the abbreviation of adjusted odds ratio.

c CI is the abbreviation of confidence interval (Adjusted OR, CI, and *p*-value per multiple logistic regression model with medical practice as the outcomes).

## 4. Discussion

Obstructive sleep apnea is a common medical condition among the general population with serious medical, psychological, and socioeconomic morbidities, yet most affected patients with OSA remain undetected and lack timely intervention. According to clinical studies, OSA causes significant adverse health and quality of life consequences and has a major impact on the global disease burden (10, 11). Despite the availability of effective diagnostic methods and treatments, screening and case identification of OSA remain difficult challenges (29). Lack of initiative to seek medical help in symptomatic patients was a barrier that impeded the early recognition of and interventions for OSA. Therefore, this lack stimulates the need for improved access to healthcare education among the general population. At the same time, experts in public health and epidemiology are also cooperating with clinicians in popular healthcare science, hoping to identify more subhealthy and preclinical patients through various forms of information provision. Early recognition and intervention for these patients will substantially lower morbidity and mortality rates and reduce overall medical costs (30). In this study, most participants showed insufficient knowledge of OSA, and their average awareness rates in several sections were <60%. Approximately nine-tenths of them had positive attitudes and practices toward OSA, especially parents. If their children had symptoms of OSA, people were more likely to undertake positive medical practices compared with themselves (95.6 vs. 87.6%). For further correlations, household economic factors and related medical history significantly influenced the medical practice pattern. None of the specific sections in scores of knowledge about OSA showed a positive effect on the medical practice pattern. The majority of respondents reported a desire to receive healthcare education through the new multimedia approach, and the majority were concerned about the etiology of OSA.

The survey focused on the general population in China, while the respondents were concentrated in the southwest and coastal areas, and the majority came from urban areas. Encouragingly, more than half of the respondents said that they heard of OSA, and only a few reported being unaware of it. The vast majority considered OSA a serious disease that warranted recognition and early intervention (76.0%). This finding suggested that OSA had a certain public awareness, and its prevalence attracted some people's attention. In the section on knowledge, only seven out of 10 respondents were able to correctly identify the four descriptions of OSA disease. In particular, patients with OSA can present drowsiness, excessive daytime sleepiness, and fatigue proneness during driving, increasing the risk of vehicular accidents (27). Although this association has been fully proven in many large-scale clinical research studies, 31.5% of people did not recognize it. In general, knowledge of OSA was not sufficient since the average awareness rate was <60%.

Among the common symptoms of OSA, “children's bed-wetting” and “nocturia >1 time/night” were the most underestimated. These two presentations are common problems in children (31, 32), affecting approximately 20% of 5-year-old children and 10% of 7-year-old children (6). Parents had a common belief that bed-wetting in children would disappear or be cured with age. However, some clinical trials have indicated that children affected by OSA were at increased risk for nocturnal enuresis (33). In addition, “nocturia > 1 time/night” is not uncommon in middle-aged and older men, who present with an increased number of nocturia episodes due to benign prostatic hyperplasia (34). Attributed to their poor predictive value, these signs have been widely ignored by the general population with potential clinical impacts. Snoring in sleep, the most common symptom recognized by 74.1% of respondents, is the chief complaint of patients with OSA. Nevertheless, this problem was always observed by patients' bedroom partners, and their complaints concerning breathing pauses triggered a referral to a sleep clinic. Regarding examinations, X-ray is the most underestimated diagnostic method for OSA. It is known that tonsil and/or adenoid hyperplasia is a major anatomical contributor to OSA in children (35). For pediatric patients, lateral radiography of the skull base is the most economical, convenient, and non-invasive examination method. Furthermore, the size of the adenoids and the degree of airway obstruction can be quantified by the N/A ratio of the nasopharyngeal airway. Therefore, it has become the first choice for clinicians to evaluate children suspected to have OSA. Compared with other knowledge sections (such as symptoms, examinations, and treatments), respondents showed lower average awareness of the complications of OSA (50.61%). This finding reflected that most people underestimated the potential risks to their health conferred by OSA. Among the complications of OSA, the most well-known were maxillofacial dysplasia (62.6%), such as adenoid face, micrognathia, dentition disorders, and protruding mouth. Owing to their obvious impact on appearance, these signs have received widespread attention among parents. During dental consultations, parents learn from the dentists that the concrete cause of OSA is adenoid/tonsil hypertrophy and receive a referral to an otolaryngologist for etiological treatment. However, spinal dysraphism (37.3%), diabetes (38.0%), and hyperlipidemia (38.8%) were commonly neglected morbidities by respondents. The relationships of OSA with endocrine, metabolic, and developmental diseases are well-documented and unequivocal (4). In addition, continuous positive airway pressure (CPAP) ventilation had the lowest rate of awareness among respondents compared with other treatments. Whereas, it is considered a first-line therapy for patients with symptomatic and moderate to severe OSA with promising clinical effects (27, 36), non-adherence and reduced compliance with CPAP have been widely seen in the literature (37, 38). The probable reasons for this question were chronic nasal congestion, intolerance to nasal masks, and the expensive price of instruments (39). Finally, the results of the present study revealed that women, those >40 years old, those with a bachelor's degree or more, and those from cities are more likely to have higher knowledge scores for OSA than other groups. This finding suggested that men, younger individuals, those with a less educated background and those from the countryside were vulnerable to OSA and required more comprehensive healthcare education.

When they had presentations about OSA, most people chose to seek medical attention for intervention. Only 12.4% (187/1507) of respondents chose not to seek medical help when they became symptomatic. The most common reason for negative medical practice was that OSA was a non-fatal disease (54.4%), indicating that the rest of the people had poor awareness of the potential risks and comorbidities. Other reasons included a lack of confidence in current therapeutic options (40.2%) and fear of surgical risks (38.2%), reflecting a lack of knowledge or clarity about the disease leading to the rejection of medical evaluation. Approximately one in five respondents reported a previous diagnosis of OSA, similar to previous reports (11). Parents displayed a more motivated attitude and practice regarding OSA for their children compared to themselves. Among parents (*n* = 1,237), 95.6% would take their children to see a doctor if symptoms or signs appeared. However, a difficult problem is that, when parents are inexperienced or unable to distinguish between normal and abnormal conditions, there is a delay in the children's condition. Surprisingly, 42.0% of parents said that their children had ever been diagnosed with OSA. In comparison to the data reported by Burghard et al. (12), that 90% of children did not receive a previous diagnosis of OSA, this population was incredibly well diagnosed. The possibility is that parents concerned about children's conditions were more likely to participate in the survey. In addition, the ratio of previously diagnosed OSA in children was higher than that in adults in this survey. This finding suggests that people are more motivated to seek medical treatment for pediatric OSA, which might be because parents are concerned about cosmetic defects in children. In terms of accepted interventions for childhood OSA (*n* = 519), only 28.1% of children had undergone surgical treatments. Although many clinical trials have strongly proved that the impaired function of children with OSA would improve after adenotonsillectomy (33, 40), most parents tend to choose a conservative policy. The reason for not considering surgical treatment might be that parents are worried that the removal of immune organs, such as tonsils or adenoids, would affect children's immunity. Another possibility is that they expect the spontaneous regression of OSA during the adolescent period since the adenoid atrophy is from 10 to 12 years old (41).

Almost nine in 10 (89.4%) respondents indicated that they were willing to receive health education. The etiology of OSA was a leading concern (74.9%), and knowledge about surgery, risks, and effects was secondary (68.2%). On the one hand, parents probably want to identify abnormalities in the early stage by learning the etiology and risk factors of OSA, such as obesity and genetic micrognathia. On the other hand, this is a possibility that there are more and more multi-child families in China with the introduction of the two-child policy, and parents are trying to prevent other children from OSA. The second hot spot is knowledge, risks, and effects of surgery, which may be a desperate consideration for solving health problems in time. In the blank of the “others” option, some parents also raised questions regarding how to distinguish between normal and abnormal snoring and whether there is an effective way to prevent OSA. These concrete data provided evidence to devise appropriate popular healthcare content. As Sia et al. (3) reported, traditional media were the most common sources of information about OSA in Singapore among the general population, followed by knowing an affected patient. In contrast, the most popular approach is the new multimedia approach (68.3%), followed by health education manuals (67.0%) in this study. With the rapid development of information technology, medical services have progressed from the traditional face-to-face mode to the current diversified medical mode. Especially after the outbreak of the COVID-19 epidemic, online outpatient clinics have rapidly increased in China (42, 43), making medical services more convenient and accessible (44). At the same time, the popular health science service does not adhere to traditional forms (such as books, brochures, and offline lectures) while creating a new internet media model with more efficient propagation and attracting content (such as short videos, interesting pictures, and WeChat subscription). These analytical outcomes suggest an increasing trend in the general population toward screening health risks by themselves, hoping to recognize abnormal conditions to prevent the occurrence and progression of the disease.

Sia et al. (3) reported that age, race, income, and education level were significant influencing factors for the ability to correctly define OSA. A Pomerania population-based survey conducted by Krüger et al. (45) in 2022 found that socioeconomic factors had no significant effects on OSA. In this survey, two regression models were established to determine the factors affecting medical practice for OSA and childhood OSA. It was illustrated that education background, household income level, place of residence, medical history, and knowledge level of OSA significantly influenced the practice of seeking medical help. For parents, work status, relationships with children, children's previous diagnosis with OSA, and overall OSA awareness were independent influencing factors for whether they would take children to the clinic. Although Krüger et al. (45) indicated that socioeconomic factors were not significantly correlated with the occurrence of OSA, the analysis in this survey confirmed the point that socioeconomic factors could modulate healthcare services. Those people who had a higher education level, household income, and lived in the city were more likely to have a positive attitude and practice toward OSA. Knowledge levels and attitudes showed predictive value for medical practice.

The theory of KAP suggests that correct understanding of diseases, developing positive attitudes, and forming healthy practices are three continuous processes of health education. In this study, the general population had a certain level of knowledge about OSA, but it was not sufficient, and positive practices were not forming completely. This outcome highlights expediting the promotion of popular science propaganda to raise awareness of OSA among the general population. As a consequence, their attitude and practices for seeking medical help could be transformed into an active mode in which the three sections promote each other. Correspondingly, the health impact on individuals and the public health burden on society would be relieved as well.

### 4.1. Limitations

As with any survey-based research, some possible limitations should be noted in this study. (1) Due to the limited objective conditions, there might be selection bias among participants due to the lack of randomization. Most of the data were collected from most areas of China without even and comprehensive distribution; therefore, the results might not represent the entire population well. (2) Urban residents and women with higher education levels were more likely to respond to the questionnaire, which might be cautiously generalized. (3) Although the researchers invited all general adult population who met the included criteria as much as possible, participation was voluntary. Therefore, people who volunteered to participate in this survey might have been more interested in healthcare knowledge than their counterparts. As mentioned above, the results need to be extrapolated prudently, especially in less educated groups and less economically developed areas. In these groups and areas, e.g., the insufficient KAP level due to OSA might be more severe. Nonetheless, data concerning the KAP and popular healthcare demand regarding OSA from nationwide samples in China supplemented the current sleep medicine studies.

## 5. Conclusion

The current study indicated that even though the higher educated and urban populations in China had a certain knowledge and positive attitudes about OSA, it was still insufficient. When symptoms or signs appeared, their limited awareness of complications and potentially severe adverse consequences hindered their inner motivation to undertake positive practice for it. The household income level, place of residence, medical history, and attitudes toward healthcare education can significantly affect people's decision to seek medical help. For parents, work status, relationships with children, and children's medical histories can be the influencing factors regarding medical practice. Economically developed factors and disparities between urban and rural areas seem to be the key points influencing the medical practice pattern regarding OSA in populations. However, people's knowledge level about OSA had no specific influence on these patterns. The majority of people expressed active demand for popular healthcare education about OSA. Making full use of the new multimedia approach to expedite the provision of popular science information could be a promising way to improve medical practice in sleep medicine among the general population.

## Data availability statement

The original contributions presented in the study are included in the article/Supplementary material, further inquiries can be directed to the corresponding author.

## Ethics statement

The studies involving human participants were reviewed and approved by the Ethics Committee on Biomedical Research of the West China Hospital of Sichuan University (Approval number: 2022.416). The patients/participants provided their written informed consent to participate in this study.

## Author contributions

DL and ZP designed the research. TM reviewed the questions. QZ, TX, QR, and TL assisted in the process of collecting data. ZP and TM conducted the data analysis. ZP wrote the manuscript. DL edited and reviewed the manuscript. All authors contributed to the article and approved the submitted version.
