# Supplementary material for: People's knowledge, attitudes, practice, and healthcare education demand regarding OSA: a cross-sectional study among Chinese general populations
Source: Front Public Health. 2023 Jul 13;11:1128334. doi: 10.3389/fpubh.2023.1128334 (PMC10372425; doi:10.3389/fpubh.2023.1128334)
Supplement: Supplementary file 2 [file Data_Sheet_2.DOCX]

Supplementary Material

People’s Knowledge, Attitudes, Practice and Health Care Education Demand Regarding OSA: A Cross-Sectional Study Among Chinese General Populations

Zhongjing Pan ^1^, Tianpei Ma ^2^, Qinghan Zeng ^1^, Ting Xu ^1^, Qiong Ran ^3^, Tianming Li ^4^, Dan Lu^1*^

*** Correspondence:** Dan Lu M.D.,PhD danlv2008@wchscu.cn

# Supplementary Data 1. Questionnaire

**I.Basic Information**

1. What is your gender?
2. Male
3. Female
4. What is your age? ___ years
5. What is your level of education?
6. College degree and below
7. Bachelor’s degree
8. Master’s degree
9. Doctoral degree

4. What is your current employment status?

A. Employed (including flexible employment)

B. Unemployed (skip to question 6)

5. What is your occupation?

A. Related medicine

B. No related medicine

6. What is your monthly household income?

A. <1,000 yuan

B. 1,000-5,000 yuan

C. 5,000-10,000 yuan

D. 10,000-30,000 yuan

E. 30,000-50,000 yuan

F. >50,000 yuan

7. What is your place of residence?

A. City

B. Countryside

8. Do you have children?

1. Yes
2. No (skip to question 11, and skip question 25,26,27,29,30 and 31)

9. What is the relationship between you and your child?

A. Mother

B. Father

C. Grandparents

D. Other:

10. How many children ≤14 years old do you have? ___ individuals

**II.Understanding of the Knowledge of Obstructive Sleep Apnea**

1. Have you ever heard of obstructive sleep apnea before?
2. None
3. Heard of it
4. Know little
5. Know more
6. Know very well

12. Whether you regard obstructive sleep apnea as a serious disease?

A. Yes

B. No

C. I don’t know

1. "Obstructive sleep apnea syndrome increases the incidence of traffic accidents":

A.True B.False C. I don’t know

1. "The most common cause of obstructive sleep apnea in children is hypertrophy of the tonsils and adenoids":

A.True B.False C. I don’t know

1. "Children's hearing loss may be related to adenoid hypertrophy":

A.True B.False C. I don’t know

16. Which picture shows an "adenoid face" due to adenoid hypertrophy?

A．
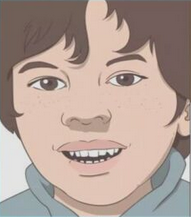
 B.
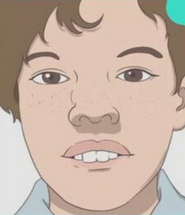
 C. I don’t know

17. How well do you know about the diagnostic methods of obstructive sleep apnea?

1. Not at all
2. Somewhat
3. Moderate
4. Quite good
5. Very well

18. Do you think obstructive sleep apnea is accompanied by the following symptoms?

| **Symptoms** | **Yes** | **No** | **Don’t know** |
| --- | --- | --- | --- |
| Sleep snoring |  |  |  |
| Apnea |  |  |  |
| Mouth breathing |  |  |  |
| Suffocation |  |  |  |
| Dyspnea |  |  |  |
| Morning Headaches |  |  |  |
| Daytime sleepiness |  |  |  |
| Susceptible to fatigue |  |  |  |
| Irritability |  |  |  |
| Nocturia >1 time /night |  |  |  |
| Children's bed-wetting |  |  |  |
| Attention deficits |  |  |  |
| Cognitive dysfunction |  |  |  |

19. Do you think the following options are common tests for diagnosing obstructive sleep apnea?

| **Examination Methods** | **Yes** | **No** | **Don’t know** |
| --- | --- | --- | --- |
| X-ray |  |  |  |
| Head CT |  |  |  |
| Nasal endoscopy |  |  |  |
| Flexible nasopharyngoscopy (FNP) |  |  |  |
| Polysomnography (PSG) |  |  |  |

20. Do you think obstructive sleep apnea increases the risk of developing the following conditions?

| **Complications** | **Yes** | **No** | **Don’t know** |
| --- | --- | --- | --- |
| Stroke |  |  |  |
| Diabetes |  |  |  |
| High blood pressure |  |  |  |
| Dementia |  |  |  |
| Arrhythmia |  |  |  |
| Myocardial infarction |  |  |  |
| Hyperlipidemia |  |  |  |
| Growth retardation |  |  |  |
| Secretory otitis media |  |  |  |
| Rhinitis/Sinusitis |  |  |  |
| Maxillofacial dysplasia |  |  |  |
| Spinal dysplasia |  |  |  |

21. How well do you know about the treatment of obstructive sleep apnea？

1. Not at all
2. Somewhat
3. Moderate
4. Quite good
5. Very well

22. Do you think the following options are the appropriate treatment for obstructive sleep apnea?

| **Treatments** | **Yes** | **No** | **Don’t know** |
| --- | --- | --- | --- |
| Weight control |  |  |  |
| Change sleep position |  |  |  |
| Medical treatment |  |  |  |
| Tonsillectomy |  |  |  |
| Adenoidectomy |  |  |  |
| Palatopharyngoplasty |  |  |  |
| Uvula shortening |  |  |  |
| Non-invasive continuous positive pressure ventilation (CPAP) / (non-invasive ventilator) |  |  |  |
| Oral appliance |  |  |  |

**III. Attitudes and Practices on Obstructive Sleep Apnea**

23. If you have symptoms such as prolonged snoring, mouth breathing, daytime sleepiness, fatigue, etc., will you go to see doctors for help?

1. Yes
2. No (skip to question 25)

24. Which department/departments do you go to? (indefinite multiple choices)

A. Respiratory medicine

B. Department of otolaryngology head and neck surgery

C. Stomatology

D. Other:

25. If your child has symptoms such as prolonged snoring and mouth breathing, will you take your child to seek for treatment?

1. Yes
2. No (skip to question 27)

26. Which department/departments will you take your child to? (indefinite multiple choices)

A. Pediatrics

B. Respiratory medicine

C. Department of otolaryngology head and neck surgery

D. Stomatology

E. Other:

27. Why would you not go to the doctor/ not take the child to the doctor? (indefinite multiple choice question)

A. During the epidemic of COVID-19, worry about the infection.

B. Obstructive sleep apnea is a non-fatal disease.

C. Lack confidence for existing treatment options.

D.Worry about surgery risks.

E. Worry about medical cost.

F. Have no time to see doctors due to the busy work.

G.Worry about the time costs affecting children's learning

H. It is inconvenient to go to the hospital

I. Other:

28. Have you ever been diagnosed with obstructive sleep apnea?

1. Yes
2. No

29. Has your child ever been diagnosed with obstructive sleep apnea?

1. Yes
2. No (skip to question 32)

30. Has your child been treated for obstructive sleep apnea?

A. Yes, the child had conservative treatment (like lifestyle changes, medication, etc.) (skip to question 32)

B. Yes, the child had underwent the surgical treatment.

C. No, the child had not been treated. (skip to question 32)

31. What surgery your child had underwent for obstructive sleep apnea? (indefinite multiple choices)

A. Tonsillectomy

B. Adenoidectomy

C. Tonsillectomy + adenoidectomy

D. Tympanocentesis/cannulation

E. Other:

**IV. Health Education Needs**

32. Would you like to receive health education about obstructive sleep apnea?

1. Yes
2. No (End the survey)

33. What would you like to hear about obstructive sleep apnea health education? (indefinite multiple choice question)

A. Causes, symptoms, complications, and treatment of obstructive sleep apnea

B. Surgery-related knowledge (surgical risks, surgical outcomes)

C. Surgical expenses and medical insurance reimbursement

D. Knowledge of perioperative nursing (preoperative preparation, postoperative precautions, postoperative pain relief methods)

E. Other:

34. Where would you like to gain knowledge about obstructive sleep apnea? (indefinite multiple choice question)

A. Traditional media (newspapers, TV, books and magazines)

B. Internet new media (Xiaohongshu, Douyin, WeChat official account, etc.)

C. Informed by medical staff when seeing the doctors

D. Health brochures produced by hospital/medical staff

E. Offline health education lectures

F. Popular science videos played in the waiting area/ward/posting posters

G. Others:

# Supplementary Data 2. Table

**Table S1. The distribution of respondents in China (N=1507)**

| Province | N | Percentage (%) |
| --- | --- | --- |
| Sichuan | 878 | 58.3 |
| Zhejiang | 188 | 12.5 |
| Jiangsu | 105 | 7 |
| Liaoning | 70 | 4.6 |
| Fujian | 66 | 4.4 |
| Shandong | 41 | 2.7 |
| Anhui | 36 | 2.4 |
| Chongqing | 21 | 1.4 |
| Guangdong | 18 | 1.2 |
| Shaanxi | 15 | 1 |
| Henan | 13 | 0.9 |
| Hebei | 13 | 0.9 |
| Yunnan | 7 | 0.5 |
| Hubei | 6 | 0.4 |
| Shanghai | 4 | 0.3 |
| Beijing | 3 | 0.2 |
| Tianjin | 3 | 0.2 |
| Ningxia | 3 | 0.2 |
| Jiangxi | 2 | 0.1 |
| Hunan | 2 | 0.1 |
| Heilongjiang | 2 | 0.1 |
| Guangxi | 2 | 0.1 |
| Hainan | 2 | 0.1 |
| Shanxi | 2 | 0.1 |
| Qinghai | 2 | 0.1 |
| Tibet | 1 | 0.1 |
| Guizhou | 1 | 0.1 |
| Inner mongolia | 1 | 0.1 |
